# Acupuncture for the Postcholecystectomy Syndrome: A Systematic Review and Meta-Analysis

**DOI:** 10.1155/2020/7509481

**Published:** 2020-07-30

**Authors:** Zihan Yin, Qiwei Xiao, Guixing Xu, Ying Cheng, Han Yang, Jun Zhou, Yanan Fu, Jiao Chen, Ling Zhao, Fanrong Liang

**Affiliations:** ^1^School of Acu-Mox and Tuina, Chengdu University of Traditional Chinese Medicine, Chengdu, China; ^2^Acupuncture Clinical Research Center of Sichuan Province, Chengdu, China

## Abstract

**Background:**

Postcholecystectomy syndrome (PCS) has become a common postoperative syndrome that requires systematic and comprehensive therapy to achieve adequate clinical control. Acupuncture and related therapies have shown clinical effects for PCS in many studies. However, systematic reviews/meta-analyses (SRs/MAs) for them are lacking.

**Objective:**

To evaluate the efficacy and safety of acupuncture in the treatment of PCS using randomized controlled trials (RCTs).

**Methods:**

Potentially eligible studies were searched in the following electronic databases up to 1 February 2020: PubMed, Embase, Cochrane Library, Web of Science (WoS), Chinese databases (Chinese Biomedical Literature Database (CBM), China National Knowledge Infrastructure (CNKI), WanFang Database (WF), and China Science and Technology Journal Database (VIP)), and other sources (WHO ICTRP, ChiCTR, Clinical Trials, and Grey Literature Database). The RevMan 5.3 was employed for analyses. The Cochrane Collaboration' risk of bias tool was used to assess the risk of bias (ROB). The Grading of Recommendations Assessment, Development, and Evaluation (GRADE) approach was used to assess the quality of the evidence.

**Results:**

A total of 14 RCTs with 1593 participants were included in this SR. MA showed that acupuncture in combination with conventional medicine (CM) did not show statistical differences in reduction in pain. However, acupuncture in combination with CM significantly reduced the incidence of postoperative nausea and vomiting (PONV) (RR, 0.71; 95% CI, 0.55–0.92) and improved gastrointestinal function recovery compared to the CM group. Acupuncture combined with traditional Chinese medicine and CM, and acupuncture as monotherapy may improve gastrointestinal function recovery with acceptable adverse events.

**Conclusion:**

Acupuncture may be an effective and safe treatment for PCS. However, this study lacks conclusive evidence due to poor quality evidence, limited data, and clinical heterogeneity of acupuncture methods in the included studies.

## 1. Introduction

Cholecystectomy is one of the surgical procedures commonly performed to treat gallbladder disease [1]. Previous studies indicate that there are approximately 700,000 cases of cholecystectomy performed each year [2]. Cholecystectomy may sometimes fail to relieve symptoms and may contribute to adverse events [3]. Postcholecystectomy syndrome (PCS) develops weeks to months after cholecystectomy and describes the presence of symptoms including abdominal pain, vomiting, and gastrointestinal symptoms [4–8]. Meanwhile, with the advent of the laparoscopic era, the number of cholecystectomy surges and the number of PCS patients may also increase [9]. The incidence of PCS has been reported to be at least 15%, and the onset of symptoms ranges from days to years [8, 10]. It seriously affects the quality of life of patients and also accelerates the deterioration of patients' diseases; so, the demand for treatment is increasing.

PCS management is challenging due to the different etiology of PCS symptoms, hence requiring specific treatment strategies. Conservative treatment and surgical therapy are the widely used treatment modalities for PCS [9]. Surgical treatment is recommended when conservative treatment is ineffective and the risk of reoperation is higher than that for the initial operation. And conservative treatment is still generally recommended. In China, acupuncture therapy (AT), an ancient conservative therapy, plays an important role in the treatment of PCS [11]. In traditional Chinese medicine (TCM), AT is used to regulate disharmony of the organ system and is theoretically used to resolve symptoms by stimulating acupuncture points related to the organs [12–14]. Meanwhile, acupuncture is effective and safe for pain [15–17], postoperative nausea and vomiting (PONV) [18, 19], and gastrointestinal function [20, 21]. Based on the evidence, the acupuncture method may be safe and effective for PCS.

However, to the best of our knowledge, there are no reported systematic reviews and meta-analysis of randomized controlled trials (RCTs) examining the efficacy and safety of acupuncture for PCS. Therefore, this study is carried out to evaluate the current evidence on the efficacy and safety of acupuncture for PCS. The Grading of Recommendations Assessment, Development, and Evaluation (GRADE) approach [22] was used to assess the quality of evidence level. We hope to provide evidence for clinical applications and references for future scientific and clinical research.

## 2. Methods

This systematic review had been registered with PROSPERO under registration number CRD42019129287 and the protocol published [23]. The study was performed based on the Preferred Reporting Items for Systematic Review and Meta-Analysis (PRISMA) guidelines [24] and A Measure Tool to Assess Systematic Reviews-2 (AMSTAR-2) [25].

### 2.1. Search Strategy

Two reviewers (QX and HY) independently conducted a comprehensive search on 4 English electronic databases (Web of Science, PubMed, Embase, and Cochrane Library), 4 Chinese electronic databases (Chinese Biomedical Literature Database (CBM), China National Knowledge Infrastructure (CNKI), WanFang Database (WF), and China Science and Technology Journal Database (VIP)), and additional sources (Grey Literature Database, WHO ICTRP, Clinical Trials, and ChiCTR) from the inception date to 1 February 2020 for potentially eligible studies. Additional trials were identified from the list of all relevant publications. The included studies were all RCTs with no language restriction.

The following search terms were used: (1) clinical condition: postcholecystectomy syndrome, cholecystectomy, cholecystotomy, and cystectomy; (2) acupuncture terms: acupuncture therapy, acupuncture-moxibustion, meridian, electro-acupuncture, acupoint, acupuncture points, acupressure-acupuncture therapy, warm needling, moxa needle, acupuncture plus moxibustion, moxibustion with warming needle, auricular acupuncture, auricular needle, ear acupuncture, moxibustion, and abdomen acupuncture; and (3) study type (randomized controlled trial). We used “and” and “or” to connect the search terms. The search strategy for PubMed is shown in Table 1 as an example.

### 2.2. Inclusion and Exclusion Criteria

#### 2.2.1. Type of Study

We included RCTs that evaluated the safety and efficacy of AT for PCS. Parallel and crossover studies were included. Nonrandomized clinical studies, quasi-RCTs, cluster RCTs, and case reports were excluded.

#### 2.2.2. Types of Participants

We included patients with PCS above the age of 18 years, regardless of race, gender, and region. We considered actual clinical conditions; however, there were no exact diagnostic criteria for PCS, but the diagnosis was confirmed based on surgery history and associated symptoms (pain, PONV, and gastrointestinal symptoms).

#### 2.2.3. Types of Interventions

We included studies in which different types of acupuncture (manual acupuncture (MA), electronic acupuncture (EA), acupuncture-moxibustion (AM), and the like) were used in the intervention group, regardless of the treatment duration and frequency. The control group included no treatment group, placebo group, pharmacological therapy, and other conventional medicine (CM) groups.

#### 2.2.4. Types of Outcome Measures

(1) *Primary Outcomes*. Pain intensity (the results measured were converted to the 11-point Numerical Rating Scale) [26] and PONV incidence [27] were analyzed.

(2) *Secondary Outcomes*. (1) The recovery of gastrointestinal function (first defecation time, first flatus time, and first bowel sounds time) [28]. (2) Adverse effects (relevant symptoms caused by acupuncture).

### 2.3. Study Selection and Data Extraction

**Figure 1:**
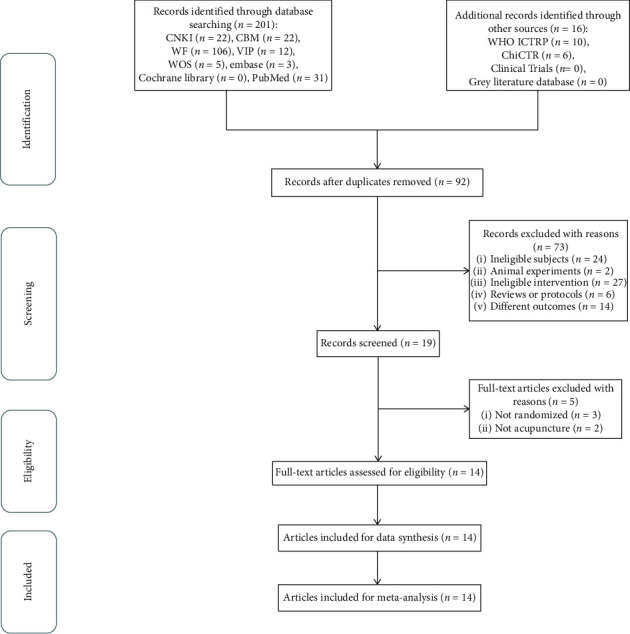
The PRISMA flowchart of selection process.

All reviewers had previous professional training on study selection and data extraction. After the elimination of duplicate studies and uploading of eligible RCTs into NoteExpress, two researchers (QX and HY) independently screened the titles, abstracts, and keywords to identify studies that potentially met the inclusion criteria. Disagreements were resolved through a discussion between the two researchers. However, if the discussion did not resolve the disagreement, a third researcher (LZ or FL) was consulted to assist in making the final decision. Details of the selection procedure for studies are shown in a PRISMA flowchart (Figure 1).

Data extraction was independently conducted by two authors (QX and HY) using standardized tabulation. The extracted information included the first author, publication date, country, sample size, mean age, gender, details of the treatment group and control group, outcome, conclusion, and acupuncture details. In case of a disagreement, a 3^rd^ party's opinion was sought to assist in making the final decision. The primary authors were contacted if any missing or additional information was needed.

### 2.4. Quality Assessment

Two authors (ZY and GX) independently assessed the risk of bias (ROB) of the included studies using the Cochrane Handbook for Systematic Reviews of Interventions [29, 30]. The following 7 items were assessed: random sequence generation, allocation concealment, blinding of participants and personnel, blinding of outcome assessors, incomplete outcome data, selective reporting, and other sources of bias. Each item was evaluated and categorized as “low risk,” “unclear,” and “high risk,” The arbiter (FL) solved any disagreement between the two reviewers.

### 2.5. Statistical Analysis

We used endpoint scores or prepost differences as outcome measures for each included study. Review Manager (RevMan) Version 5.3 software (Cochrane, London, UK) suggested by Cochrane Collaboration was used for quantitative synthesis. For the meta-analyses, the fixed-effects model by the Mantel–Haenszel method was used; otherwise, the random-effects model adopted by the DerSimonian–Laired method was used. The *I*^2^ statistic was used to measure the heterogeneity among the studies. We considered that there was no heterogeneity when *p* > 0.1 and *I*^2^ < 50%, and all data were analyzed with 95% CIs. The dichotomous data were analyzed by risk ratios (RR), while for continuous data, the standard mean differences (SMD) were used. The subgroup was adopted on the condition of high data heterogeneity. Funnel plots were used to measure publication bias when the number of included studies was more than 10. If the funnel chart was evenly distributed, there was no reporting of bias.

### 2.6. Quality of Evidence

The GRADE approach [22] was used by the reviewers to assess the quality of evidence of the obtained outcome indicators from five items of research limitations, inconsistency, indirectness, inaccuracy, and publication bias. The quality of evidence was rated as “very low,” “low,” “moderate,” or “high” based on GRADE rating standards. The quality of the evidence “high” indicates that future research is very unlikely to change existing evidence; “moderate” indicates that future research may change the results; being “low” level indicates that future research is likely to have an important impact on existing evidence and is likely to change the evaluation results; and “very low” indicates that we are highly uncertain about the existing evidence.

## 3. Results

### 3.1. Study Description

#### 3.1.1. Literature Search

In the initial stage of selection, 162 Chinese studies and 39 English studies were collected, and 16 data were obtained from other sources. After excluding 125 duplicate literatures, 92 RCTs remained. By the end of initial screening, there were 19 studies left. Finally, 5 studies were excluded (2 non-RCT and 3 nonacupuncture) and 14 studies remained [31–44]. The PRISMA flowchart is shown in Figure 1, and full-text articles excluded with reasons are covered in Appendix B.

#### 3.1.2. Study Characteristics

Table 2 presents the characteristics of the included RCTs. Among the 14 [31–44] included RCTs, 1 [31] was in Turkish and 13 [32–44] were in Chinese. The RCTs were published from 2006 to 2019, and they were reported in full-texts. All patients were adults (age >= 18 years old). A total of 14 RCTs with 1593 participants, 870 in the intervention group and 723 in the control group, were included in this systematic review and meta-analysis. Experimental interventions included manual acupuncture (MA), acupuncture-moxibustion (AM), and electroacupuncture (EA). The control groups received conventional medicine (CM), which included conventional nutrition rehydration and anti-infective agents, while some studies reported the use of tramadol [31], cisapride [34], ondansetron [37], metoclopramide [39], morphine [41, 43], fentanyl [43], and other interventions. As outcome measures, the recovery of gastrointestinal function (first defecation time, 1^st^ flatus time, and 1^st^ bowel sounds time) [32, 33, 38, 40, 44] was the most mentioned primary outcome measure, and the change of pain intensity [31, 35, 36, 41, 43] was evaluated in 5 trials.

#### 3.1.3. Acupuncture Details

MA was reported in most studies [31, 35, 37, 39, 40, 42, 43], EA was used in 5 studies [32, 33, 36, 41, 42], and AM was used in 2 studies [34, 38]. Zu San Li (ST36) [32–44] was the most frequently used acupoints, while Nei Guan (PC6) [31, 32, 34–39, 43] was reported to be used in 9 trials. The reported insertion depth was 0.25 mm–40 mm, and the penetration depth varied widely due to the use of different acupoints. A total of 9 studies [31, 34–39, 42, 44] were exposed to deqi, and the 9 RCTs [31, 32, 36, 37, 39–42, 44] reported needle stimulation. The most commonly used needle retention time was 30 minutes; the most frequent number of treatment sessions was 3; and the most commonly used duration and frequency of AT treatment was 72 hours and 1-2 times/day. Details of the acupuncture therapies used are shown in Table 3.

### 3.2. Quality Assessment

**Figure 2:**
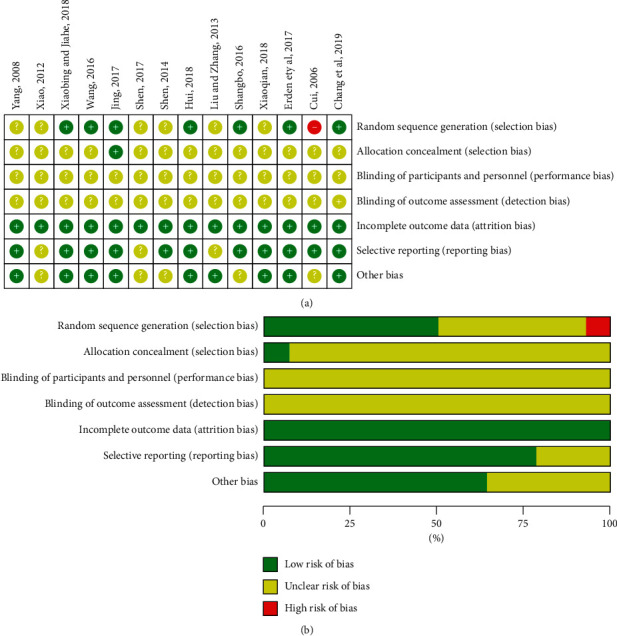
(a) Risk of bias summary. (b) Risk of bias graph.

All the 14 trials included were described as RCTs. We measured the ROB by Cochrane Handbook V.5.3.0. The use of random sequence generation was reported in 7 studies [31, 32, 35, 36, 40–42], out of which one RCT [33] had “high risk,” and the descriptions in 6 [34, 37–39, 43, 44] studies were unclear; allocation concealment was assessed as being “low risk” in 1 study [40], while other [31–39, 41–44] RCTs did not report allocation concealment; no study mentioned blinding of participants and outcome assessors; all studies [31–44] indicated that the outcome data were complete and were assessed as “low risk;” in selective outcome reporting, 11 studies [31–36, 39–42, 44] were assessed as “low risk” and 3 RCTs [37, 38, 43] were assessed as “unclear” due to lack of sufficient information; and in the bias category, 9 studies [31, 32, 34, 36, 37, 40–42, 44] were ranked to be at “low risk” and 5 RCTs [33, 35, 38, 39, 43] were judged as “unclear” due to lack of adequate information.  Figure 2 presents a summary of the ROB for each included study.

### 3.3. Effects of Intervention

The summaries for all comparison results and GRADE analyses are shown in Table 4. There was great heterogeneity, and we had performed a subgroup analysis based on the type of acupuncture.

#### 3.3.1. Reduction in Pain Intensity

Based on the existing strong correlation between the pain assessment scales, the visual analogue scale (VAS), or other scales, results were all converted to the 11-point digital rating scale (0 points for no pain, and 10 points for the most severe pain) [45].

**Figure 3:**
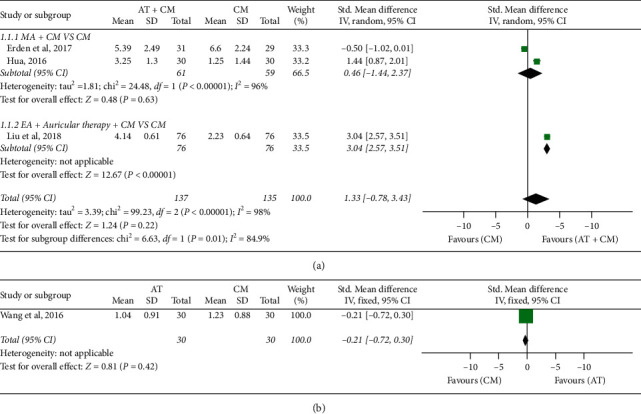
Forest plot of reduction in pain intensity: (a) AT + CM vs. CM and (b) AT vs. CM.

*(1) AT* + *CM versus CM.* There were no statistical differences reported between AT + CM and CM results (*n* = 272; SMD, 1.33; 95% CI, −0.78 to 3.43; *p*=0.22; heterogeneity: *X*^2^ = 99.23, *p* < 0.00001, *I*^2^ = 98%). In subgroup analyses, MA + CM and CM showed no statistically significant differences (*n* = 100; SMD, 0.46; 95% CI, −1.44 to 1.37; *p*=0.63; Figure 3(a)). EA + auricular therapy + CM and CM showed statistically significant differences (*n* = 152; SMD, 1.33; 95% CI, −0.78 to 3.43; *p* < 0.00001; Figure 3(a)).

Erden et al. [31] used CM and tramadol and reported no statistically significant difference between AT + CM with tramadol and CM with tramadol (*n* = 60; SMD, −0.50; 95% CI, −1.02 to 0.01; *p*=0.06). The study further reported that the application of acupuncture did not cause any change in the consumption of tramadol. We carried out sensitivity analysis, and the study was excluded and the meta-analysis repeated. The results indicated that there was a significant difference between AT + CM and CM (*n* = 212; SMD, 2.25; 95% CI, 0.68–3.82; *p*=0.005). The quality of evidence for the outcome was “low.”

*(2) AT versus CM.* There was no significant difference between AT and CM (*n* = 60; SMD, −0.21; 95% CI −0.72 to 0.30; *p*=0.42; Figure 3(b)). Wang [41] reported that there was no significant difference between acupuncture and CM combined with morphine. The quality of evidence for this outcome was “moderate”.

#### 3.3.2. POVN Incidence

The POVN effect is defined as the ratio of the number of people showing POVN after treatment to the total number of people in the treatment group.

**Figure 4:**
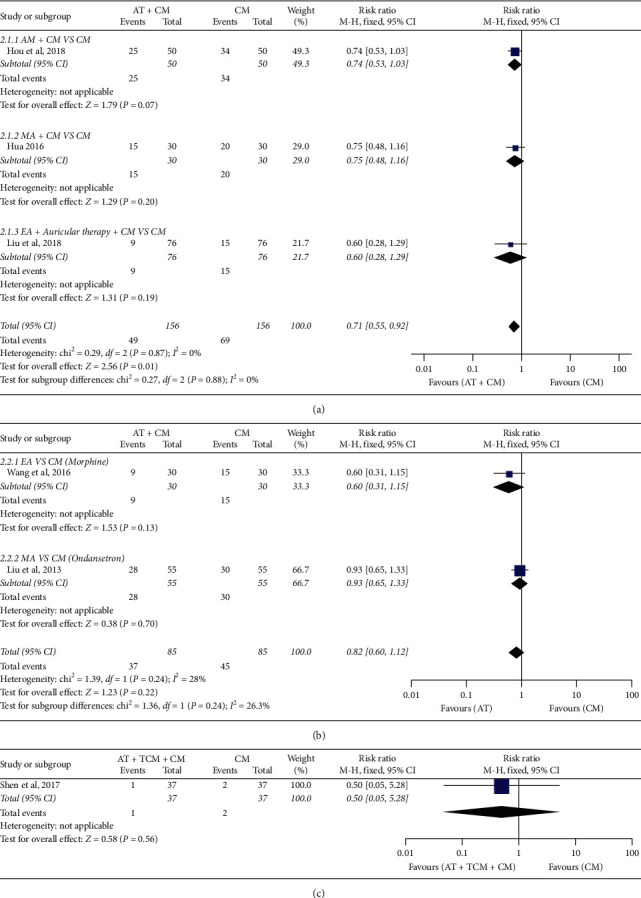
Forest plot of POVN incidence: (a) AT + CM vs. CM, (b) AT vs. CM, and (c) AT + TCM + CM vs. CM.

*(1) AT* + *CM versus CM.* Statistically significant difference was reported in POVN between AT plus CM and CM (*n* = 312; RR, 0.71; 95% CI, 0.55 to 0.92; *p*=0.01; heterogeneity: *X*^2^ = 0.29, *p*=0.87, *I*^2^ = 0%; Figure 4(a)). Subgroup analyses revealed that AM + CM and CM showed no significant differences (*n* = 100; RR, 0.74; 95% CI, 0.53 to 1.03; *p*=0.07; Figure 4(a)); MA + CM and CM had no statistically significant differences (*n* = 60; RR, 0.75; 95% CI, 0.48 to 1.16; *p*=0.20; Figure 4(a)); and EA + auricular therapy + CM and CM had no statistically significant differences (*n* = 152; RR, 0.60; 95% CI, 0.28 to 1.29; *p*=0.19; Figure 4(a)). The quality of the evidence shown was “moderate.”

*(2) AT versus CM.* There was no significant difference between AT and CM (*n* = 170; RR, 0.82; 95% CI, 0.60 to 1.12; *p*=0.22; Figure 4(b)). The quality of evidence for the outcome was “moderate.”

*(3) AT* + *TCM* + *CM versus CM.* There was no statistically significant difference between AT + TCM + CM and CM (*n* = 74; RR, 0.5; 95% CI, 0.05 to 5.28; *p*=0.58; Figure 4(c)). The quality of evidence was “very low.”

#### 3.3.3. The Recovery of Gastrointestinal Function

**Figure 5:**
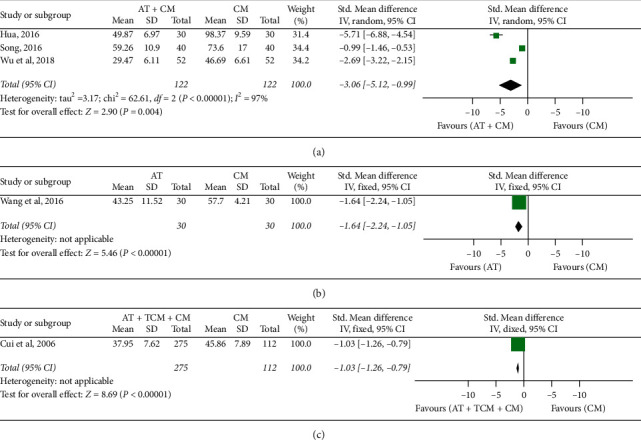
Forest plot of first defecation time: (a) AT + CM vs. CM, (b) AT vs. CM, and (c) AT + TCM + CM vs. CM.

*(1) First Defecation Time*. *AT* *+* *CM versus CM*: there was a statistically significant difference reported in first defecation time between AT plus CM and CM (*n* = 244; SMD, −2.05; 95% CI, −2.39 to −1.72; *p* < 0.00001; heterogeneity: *X*^2^ = 62.61, *p* < 0.00001, *I*^2^ = 97%; Figure 5(a)). The quality of evidence for the outcome was “moderate.” *AT versus CM*: there was statistically significant difference between AT and CM (*n* = 60; SMD, −1.64; 95% CI, −2.24 to −1.05; *p* < 0.00001; Figure 5(b)). The quality of evidence was “moderate.” *AT* *+* *TCM* *+* *CM versus CM*: there was a statistically significant difference between AT + TCM + CM and CM (*n* = 387; SMD, −1.03; 95% CI, −1.26 to −0.79; *p* < 0.00001; Figure 5(c)). The quality of evidence for the outcome was “low.”

**Figure 6:**
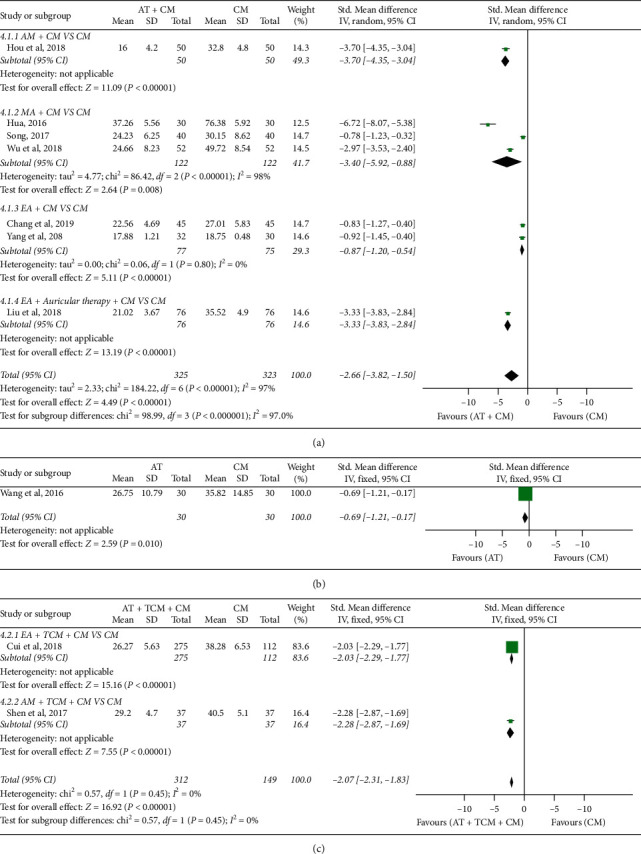
Forest plot of first flatus time: (a) AT + CM vs. CM, (b) AT vs. CM, and (c) AT + TCM + CM vs. CM.

*(2) First Flatus Time*. *AT* *+* *CM versus CM*: a statistically significant difference was shown in first defecation time between AT plus CM and CM (*n* = 648; SMD, −2.66; 95% CI, −3.82 to −1.50; *p* < 0.00001; heterogeneity: *X*^2^ = 184.22, *p* < 0.00001, *I*^2^ = 97%; Figure 6(a)). Subgroup analyses revealed that EA + CM and CM showed statistically significant differences (*n* = 152; SMD, −0.87; 95% CI, −1.20 to −0.54; *p* < 0.00001; heterogeneity: *X*^2^ = 0.06, *p*=0.80, *I*^2^ = 0%; Figure 6(a)); MA + CM and CM had statistically significant differences (*n* = 244; SMD, −3.40; 95% CI, −5.92 to −0.88; *p*=0.008; heterogeneity: *X*^2^ = 86.42, *p* < 0.00001, *I*^2^ = 98%; Figure 6(a)); AM + CM and CM showed statistically significant differences (*n* = 100; SMD, −3.70; 95% CI, −4.35 to −3.04; *p* < 0.00001; Figure 6(a)); and EA + auricular therapy + CM and CM showed statistically significant differences (*n* = 152; SMD, −3.33; 95% CI, −3.83 to −2.84; *p* < 0.00001; Figure 6(a)). The quality of evidence for the outcome was “low.” AT versus CM: there were significant differences between AT and CM (*n* = 60; SMD, −0.69; 95% CI, −1.21 to −0.17; *p*=0.01; Figure 6(b)). The quality of evidence for the outcome was “moderate.” AT + TCM + CM versus CM: there were significant difference between AT + TCM + CM and CM (*n* = 461; SMD, −2.07; 95% CI, −2.31 to −1.83; *p* < 0.00001; heterogeneity: *X*^2^ = 0.57, *p*=0.45, *I*^2^ = 0%; Figure 6(c)). Subgroup analyses revealed that EA + TCM + CM and CM showed significant differences (*n* = 387; SMD, −2.03; 95% CI, −2.29 to −1.77; *p* < 0.00001; Figure 6(c)), and AM + TCM + CM and CM showed significant differences (*n* = 74; SMD, −2.28; 95% CI, −2.87 to −1.69; *p* < 0.00001; Figure 6(c)). The quality of evidence for the outcome was “low.”

**Figure 7:**
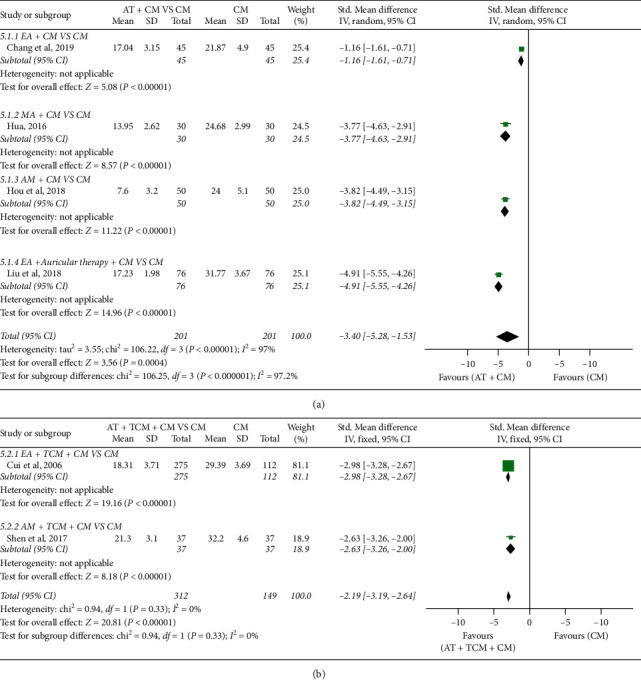
Forest plot of first bowel sounds time: (a) AT + CM vs. CM and (b) AT + TCM + CM vs. CM.

*(3) First Bowel Sounds Time*. *AT* *+* *CM versus CM*: significant differences were reported between AT + CM and CM (*n* = 402; SMD, −2.85; 95% CI, −3.15 to −2.55; *p* < 0.00001; heterogeneity: *X*^2^ = 106.25, *p* < 0.00001, *I*^2^ = 97%; Figure 7(a)). Subgroup analyses showed that EA + CM and CM had significant differences (*n* = 90; SMD, −1.16; 95% CI, −1.61 to −0.71; *p* < 0.00001; Figure 7(a)); MA + CM and CM had significant differences (*n* = 60; SMD, −3.77; 95% CI, −4.63 to −2.91; *p* < 0.00001; Figure 7(a)); AM + CM and CM had significant differences (*n* = 100; SMD, −3.82; 95% CI, −4.49 to −3.15; *p* < 0.00001; Figure 7(a)); and EA + auricular therapy + CM and CM had significant differences (*n* = 152; SMD, −4.91; 95% CI −5.55 to −4.26; *p* < 0.00001; Figure 7(a)). The quality of evidence for the outcome was “low.” AT + TCM + CM versus CM: significant differences were reported between AT + TCM + CM and CM (*n* = 461; SMD, −2.91; 95% CI, −3.19 to −2.64; *p* < 0.00001; heterogeneity: *X*^2^ = 0.94, *p*=0.33, *I*^2^ = 0%; Figure 7(b)). Subgroup analyses showed that EA + TCM + CM and CM had significant differences (*n* = 387; SMD, −2.98; 95% CI −3.28 to −2.67; *p* < 0.00001; Figure 7(b)), and AM + TCM + CM and CM had significant differences (*n* = 74; SMD, −2.63; 95% CI −3.26 to −2.00; *p* < 0.00001; Figure 7(b)). The quality of evidence for the outcome was “low.”

### 3.4. Safety

A total of 4 RCTs with 424 participants [37–40, 43] provided information on adverse events associated with acupuncture (Table 5). One trial [40] reported that no adverse events occurred during the interventions, and adverse events were reported in the other 3 studies. Only 2 events [39, 43] reported dizziness during acupuncture. A total of three studies [37, 38, 43] reported that CM could cause dizziness, constipation, extrapyramidal symptoms, PONV, and hypotension. However, based on other existing studies, acupuncture was safe for PCS. Table 5 presents the details of the adverse events.

### 3.5. Heterogeneity

Acupuncture methods, techniques, acupoints, depth of insertion, acupuncture doses, acupuncture operators, acupuncture retention duration, and treatment sessions among other factors were varied, which may lead to high clinical heterogeneity; hence, subgroup analysis was performed. Meanwhile, medication therapy showed heterogeneity on accounts of different types of drugs and dosages. However, since most of the studies did not provide adequate CM information, we did not accomplish the subgroup analyses. Finally, we found that the subgroup according to the type of acupuncture could better illustrate the heterogeneity. And we also tried to perform sensitivity analysis by excluding studies that were “high risk;” however, very few articles were included leading to high risk of bias.

### 3.6. Reporting Bias

Since the number of included studies did not exceed 10, funnel plots were not used to measure publication bias.

### 3.7. Quality of Evidence

The GRADE approach was used to evaluate the quality of the evidence of the included studies, and the analyses are presented in Table 4. Outcomes were the reduction in pain intensity, POVN incidence, 1^st^ defecation time, 1^st^ flatus time, and 1^st^ bowel sounds time. A total of 13 outcomes were applied to the RCTs. The quality of the evidence for the overall outcomes was acceptable. The results showed that there was 1 (1/13, 7.7%) outcome with very low quality evidence, 6 (6/13, 46.15%) with low quality evidence, 3 (6/13, 46.15%) with moderate quality evidence, and none with high quality evidence. However, it is difficult for therapists and patients to use blinding for acupuncture. Therefore, future research should pay more attention to the above aspects and avoid the risk of prejudice or revise evaluation tools to make them more suitable for acupuncture, Chinese medicine therapy, or other conservative treatment.

## 4. Discussion

In the current study, we conducted a systematic review and meta-analysis of 14 RCTs with 1593 participants to evaluate the efficacy and safety of acupuncture for PCS. The outcomes assessment of this review are summarized in 3 aspects: the change of pain before and after treatment, the incidence of POVN, and the recovery of gastrointestinal function (first defecation time, 1^st^ flatus time, and 1^st^ bowel sounds time). There were significant differences between acupuncture and CM in the POVN and the recovery of gastrointestinal function. However, acupuncture plus CM with single CM did not show statistical differences in reduction of pain.

When acupuncture was added to CM, our results indicated no significant reductions in pain intensity between CM and CM + acupuncture. A previous study [31] reported no significant difference in pain intensity between acupuncture + CM (tramadol) and CM (tramadol). However, the quality of evidence for this study was “low”; hence, we disputed these results. No significant differences in pain were reported between acupuncture and CM (morphine). However, only one trial [41] evaluated the differences in pain efficacy between acupuncture and CM. Therefore, the results concerning the efficacy of acupuncture as monotherapy for pain reduction in PCS should be interpreted with caution.

Even though the quality of the evidence for the outcome was moderate, 3 studies [34–36] showed significant differences between AT + CM and CM in POVN. No significant differences in efficacy were reported between AT and CM. In addition, AT + TCM + CM showed no significant differences from CM.

In recovery of gastrointestinal function, AT + CM VS CM, AT VS CM, and AT + TCM + CM VS CM showed significant differences. However, this review reported significant differences between acupuncture and CM, and the quality of the evidence for the outcome was moderate. In addition, the results indicated that acupuncture might improve first defecation time and 1^st^ flatus time; however, due to the limited number of studies included, these results require further investigations. The significant differences in efficacy reported between AT + TCM + CM and CM were not conclusive due to the low level of evidence and the limited number of studies included.

Only 4 RCTs (28.57%) reported safety data for acupuncture, and there was no reported evidence of association of acupuncture with any serious adverse events. Therefore, this review could not draw any firm conclusions on the safety of acupuncture for PCS.

This review has several limitations. (1) Despite our efforts to reduce bias and the inclusion of grey literature, we are not sure that all studies were included. (2) The number of selected studies and the sample size in most of the studies were small. (3) The low methodological quality of some RCTs remains a challenge. However, many studies showed performance bias since acupuncture was difficult to blind. The low methodological quality of some RCTs may cause overestimation of the effects of acupuncture on PCS. (4) In some studies, significant heterogeneity was reported for several outcomes. These may have been caused by a number of factors such as age, gender, and surgical methods among other factors of the recruited PCS patients. In addition, acupuncture clinical trials involve many factors that may lead to heterogeneity, such as acupoint selection, depth of insertion, deqi, needle stimulation, needle retention duration, number of treatment sessions, frequency of treatment, and duration. Even though the treatment method used in the control group is CM, the differences in dosage and dosage forms may have caused heterogeneity. (5) The quality of various outcomes evidence included mainly low and moderate quality evidence. Therefore, future research may have a significant impact on existing evidence and may change the evaluation results.

We provide prospects and suggestions for future research. Previous research reveals that PCS lacks a widely accepted diagnostic standard. Future research must standardize and generalize acupuncture treatment for PCS. It is important to consider possible clinical heterogeneity due to inconsistencies in the types of acupuncture, acupoint selection, acupuncture retention time, stimulation intensity, and course. In this review, most of the included studies used the ST 36 and PC 6 acupoints. The most frequent needle retention time was 30 minutes, and the number of treatments was 6 times. The treatment time was once a day. According to the current RCTs included in this review, the methodological quality and evidence quality was not high. Therefore, in future, a multicentered, large sample, high quality RCT should be conducted in full compliance with the Consolidated Standards of Reporting Trials (CONSORT) [46], Standards for Reporting Interventions in Clinical Trials of Acupuncture (STRICTA) [47], and Cochrane Handbook for Systematic Reviews of Interventions to control the methodological quality.

## 5. Conclusion

The results of this SR/MA indicated that acupuncture may improve the overall symptoms of PCS. The reported acupuncture-related adverse events are mild and acceptable. However, due to limited data, heterogeneity of acupuncture methods among the RCTs and the low methodological quality of some of the RCTs, there is a need for additional and well-designed RCTs with larger sample sizes to be performed to confirm these results.

## Figures and Tables

**Table 1:** Search strategy for the PubMed database.

#1 postcholecystectomy syndrome[Title/Abstract]
#2 cholecystectomy[Title/Abstract]
#3 cholecystotomy[Title/Abstract]
#4 cystectomy[Title/Abstract]
#5 #1 OR #2 OR #3 OR #4
#6 acupuncture therapy[Title/Abstract]
#7 acupuncture-moxibustion[Title/Abstract]
#8 meridian^*∗*^[Title/Abstract]
#9 electro-acupuncture[Title/Abstract]
#10 #6 OR #7 OR #8 OR #9
#11 acupoint[Title/Abstract]
#12 acupuncture points[Title/Abstract]
#13 acupressure[Title/Abstract]
#14 acupressure-acupuncture therapy[Title/Abstract]
#15 #11 OR #12 OR #13 OR #14
#16 warm needling[Title/Abstract]
#17 moxa needle[Title/Abstract]
#18 acupuncture plus moxibustion[Title/Abstract]
#19 moxibustion with warming needle[Title/Abstract]
#20 #16 OR #17 OR #18 OR #19
#21 auricular acupuncture[Title/Abstract]
#22 auricular needle[Title/Abstract]
#23 ear acupuncture[Title/Abstract]
#24 moxibustion[Title/Abstract]
#25 #21 OR #22 OR #23 OR #24
#26 abdom^*∗*^ acupuncture[Title/Abstract]
#27 embedded thread therapy[Title/Abstract]
#28 embedding thread[Title/Abstract]
#29 catgut embedding[Title/Abstract]
#30 #26 OR #27 OR #28 OR #29
#31 #10 OR #15 OR #20 OR #25 OR #30
#32 #5 AND #31
#33 Postcholecystectomy Syndrome[MeSH Terms]
#34 Acupuncture therapy[MeSH Terms]
#35 (#5 OR #33) AND #34
#36 #35 OR #32
#37 clinical[Title/Abstract]
#38 trial[Title/Abstract]
#39 #37 AND #38
#40 clinical trials as topic[MeSH Terms]
#41 clinical trial[Publication Type]
#42 random^*∗*^[Title/Abstract]
#43 random allocation[MeSH Terms]
#44 therapeutic use[MeSH Subheading]
#45 #39 OR #40 OR #41 OR #42 OR #43 OR #44
#46 #45 AND #36

**Table 2:** Main characteristics of included RCTs.

Study (reference)	Country	Sample size (A)/(B)	Mean age (A)/(B)	Gender (M : F) (A)/(B)	(A) Treatment group	(B)Control group	Acupoints	Outcomes		Conclusion (+/−)
								Primary outcomes	Secondary outcomes	
Erden et al., 2017 [31]	Turkey	31/29	46.77/45.64	A: (6 : 25) B: (3 : 25)	MA + (B)	CM (tramadol)	Ri Yue (GB24), Yang Ling Quan (GB34), Guang Ming (GB37), di Wu Hui (GB42), Xing Jian (LR2), Nei Gu (PC6), He Gu (LI4)	Postoperative pain scores (NRS)	(1) Postoperate satisfaction index (2) Analgesic consumption	Despite detection of a reduction in postoperative pain scores, the application of acupuncture did not cause any change in the consumption of tramadol (+)
Chang et al., 2019 [32]	China	45/45	(39.72 ± 5.08)/c(39.41 ± 5.25)	A: (31 : 14) B: (28 : 17)	EA + (B)	CM	San Yin Jiao (SP6), Nei Guan (PC6), Zu san Li (ST37)	The recovery of gastrointestinal function (first defecation time, 1^st^ flatus time, and 1^st^ bowel sounds time)	(1) Visual analogue rating of nausea (2) Ventriculin	Electroacupuncture can promote postoperative recovery of patients with laparoscopic cholecystectomy and regulate gastric peristalsis (+)
Cui 2006 [33]	China	275/112	39.6/40.5	A: (85 : 190)cB: (23 : 89)	EA + TCM + (B)	CM	Zu san Li (ST38)	The recovery of gastrointestinal function (first defecation time, 1^st^ flatus time, and 1^st^ bowel sounds time)	—	Acupuncture and traditional Chinese medicine can promote postoperative recovery of patients with laparoscopic cholecystectomy (+)
Xiaoqian 2018 [34]	China	50/50	(50.5 ± 7.0)/(50.3 ± 5.0)	(40 : 60)	AM + (B)	CM (cisapride)	Zhong Wan (RN12), Dan shu (BL19), Gan shu (BL18), Nei Guan (PC6), Zu san Li (ST36)	The clinical curative effect	(1) The recovery of gastrointestinal function (first defecation time, 1^st^ flatus time, and 1^st^ bowel sounds time) (2) PONV	MA can promote recovery of gastrointestinal function after cystic resection adjustment (+)
Shangbo 2016 [35]	China	30/30	(46.27 ± 6.39)/(45.72 ± 6.18)	A: (17 : 13) B: (14 : 16)	MA + (B)	CM	Gong sun (SP4), shang Ju Xu (ST37), Nei Guan (PC6), Zu san Li (ST36)	Postoperative pain scores	(1) The recovery of gastrointestinal function (first defecation time, 1^st^ flatus time, and 1^st^ bowel sounds time) (2) Ventriculin (GAS) (3) PONV	For patients undergoing cholecystectomy, acupuncture on the basis of CM therapy can achieve better efficacy than CM of patients (+)
Hui 2018 [36]	China	76/76	(37.6 ± 5.1)/(35.8 ± 8.5)	A: (41 : 35) B: (39 : 37)	EA + auricular therapy + (B)	CM	Nei Guan (PC6), He Gu (LI4), Zu san Li (ST36)	Postoperative pain scores	(1) The recovery of gastrointestinal function (first flatus time and 1^st^ bowel sounds time) (2) PONV	Electroacupuncture combined with auricular therapy can significantly improve the recovery of patients after laparoscopic cholecystectomy (+)
Liu and Zhang 2013 [37]	China	55/55	(51.4 ± 10.2)/(50.6 ± 9.7)	A: (16 : 39) B: (18 : 37)	MA	CM (ondansetron)	Nei Guan (PC6), Tian Tu (RN22), Zu san Li (ST37), Ju Que (RN14), Xia Wan (RN10), Bu Rong (ST19), Tai Yi (ST23)	PONV	Adverse events	Acupuncture is efficacy and safe in treating vomiting after laparoscopic cholecystectomy (+)
Shen 2017 [38]	China	37/37	49.3	(26 : 48)	AM + TCM + (B)	CM	Yang Ling Quan (GB34), Zu san Li (ST38), san Yin Jiao (SP6), Nei Guan (PC6)	The recovery of gastrointestinal function (first flatus time and 1^st^ bowel sounds time)	(1) PONV. (2) Adverse events	After cholecystectomy, acupuncture combined with traditional Chinese medicine can shorten the recovery time of gastrointestinal function and reduce the incidence of adverse reactions (+)
Shen 2014 [39]	China	57/57	(36.5 ± 12.7)/(35.0 ± 11.3)	A: (26 : 31) B: (25 : 32)	MA + (B)	CM (metoclopramide)	Nei Guan (PC6), Tian Tu (RN22), Zu san Li (ST37), Ju Que (RN14), Xia Wan (RN10), Bu Rong (ST19), Tai Yi (ST23)	The clinical curative effect	Adverse events	Metaclopramide and acupuncture are effective in treating PONV with minor adverse reaction (+)
Jing 2017 [40]	China	40/40	(47.23 ± 11.68)/(48.12 ± 14.47)	A: (17 : 23) B: (17 : 23)	MA + (B)	CM	Shang Ju Xu (ST37), Zu san Li (ST36), san Yin Jiao (SP6)	The recovery of gastrointestinal function (first defecation time, 1^st^ flatus time, and 1^st^ bowel sounds time)	(1) The clinical curative effect (2) Adverse events	Acupuncture is efficacy and safe in treating the recovery of gastrointestinal function after laparoscopic cholecystectomy (+)
Wang 2016 [41]	China	30/30	(53.50 ± 8.30)/(51.30 ± 8.10)	A: (13 : 17) B: (14 : 16)	EA	CM (morphine)	Zu san Li (ST37), Tai Chong (ST23), Yang Ling Quan (GB34)	Pain intensity, the nausea incidence and the vomiting incidence	The recovery of gastrointestinal function (first defecation time and 1^st^ flatus time)	Electroacupuncture could effectively relieve postoperative pain and promote the recovery of gastrointestinal function after operation, which reduced the incidence of PONV without excessive sedation (+)
Xiaobing and Jiahe 2018 [42]	China	52/52	(55.23 ± 3.4)/(56.37 ± 3.1)	A: (30 : 22) B: (24 : 28)	MA + (B)	CM	Zu san Li (ST37), Xia Wan (RN10), Zhong Wan (RN12), Guan Yuan (RN4), Hua Rou Men (ST24), Qi Hai (RN6)	The clinical effective	(1) The recovery of gastrointestinal function (first defecation time and 1^st^ flatus time) (2) Ventriculin (MMP-9, TIMP-1) (3) Quality of life	Moxibustion and combined with MA for postoperative gastrointestinal function in patients with laparoscopic cholecystectomy has good therapeutic significance (+)
Xiao 2012 [43]	China	60/60	18–78	A: (44 : 16) B: (40 : 20)	MA	CM (fentanyl and morphine)	Yang Ling Quan (GB34), He Gu (LI4), Zu san Li (ST38), san Yin Jiao (SP6), Nei Guan (PC6), Dan Nang (EX-LE6), A shi point	Postoperative pain scores	Adverse events	Acupuncture is effective in the analgesia after laparoscopic cholecystectomy and has fewer adverse reactions such as the digestive tract (+)
Yang and Liu 2008 [44]	China	32/30	(68.59 ± 2.44)/(69.97 ± 1.59)	A: (11 : 21) B: (8 : 22)	EA + (B)	CM	Zu san Li (ST38), san Yin Jiao (SP6)	The recovery of gastrointestinal function (1^st^ flatus time)	—	The treatment of acupuncture can accelerate the recovery of gastrointestinal function in patients after laparoscopic cholecystectomy (+)

**Table 3:** Details of acupuncture treatment methods.

Study (reference)	Depth of insertion	Deqi	Needle stimulation	Needle retention duration	Number of treatment sessions	Frequency of treatment	Duration
Erden et al., 2017 [31]	0.25–0.3 mm	Y	Twirling every 10 min	30 min	6	0, 1^st^, 2^nd^, 6^th^, 12^th^, and 18^th^	18 h
Chang et al., 2019 [32]	NR	NR	Electrical stimulation (30 times per minute)	20 min	NR	1 time every 4 hours	NR
Cui 2006 [33]	NR	NR	NR	20 min	4	2 times everyday	48 h
Xiaoqian 2018 [34]	20–30 mm	Y	NR	30 min	28	1 time everyday	4 w
Shangbo 2016 [35]	25–32.5 mm	Y	NR	30 min	4	2 times everyday	48 h
Hui 2018 [36]	NR	Y	Electrical stimulation (4–20 HZ)	30 min	3	1 time everyday	72 h
Liu and Zhang 2013 [37]	7.5–40 mm	Y	Twirling 1-2 times per 30 min	20–30 min	3	1 time everyday	72 h
Shen 2017 [38]	NR	Y	NR	20–30 min	3–6	1–2 times everyday	72 h
Shen 2014 [39]	7.5–40 mm	Y	Twirling every 5 min	20–30 min	3	1 time everyday	72 h
Jing 2017 [40]	NR	NR	Electrical stimulation (10 HZ)	30 min	<5	1 time everyday	<120 h
Wang 2016 [41]	40 mm	NR	Electrical stimulation (2 HZ)	30 min	2	2 times everyday	24 h
Xiaobing and Jiahe 2018 [42]	40 mm	Y	Twirling every 30 min	30 min	NR	NR	4 w
Xiao 2012 [43]	NR	NR	NR	10–15 min	9	3 times everyday	72 h
Yang and Liu 2008 [44]	NR	Y	Electrical stimulation (30 times per minute)	20 min	NR	1 time every 4 hours	NR

Notes: NR: not recorded; Y: yes.

**Table 4:** Quality of evidence included RCTs by GRADE.

Interventions	Included RCTs (patients)	Relative effect (95% CI)	Quality assessment					Quality of evidence
			Risk of bias	Inconsistency	Indirectness	Imprecision	Publication bias	
*Reducing pain intensity*								
AT + CM	3 (272)	SMD 1.33 (−0.78 to 3.43)	−1①	−1②	0	0	0	Low
AT	1 (60)	SMD −0.21 (−0.72 to 0.30)	0	0	0	−1③	0	Moderate

*POVN*								
AT + CM	3 (312)	RR 0.71 (0.55 to 0.92)	−1①	0	0	0	0	Moderate
AT	2 (170)	RR 0.82 (0.60 to 1.12)	−1①	0	0	0	0	Moderate
AT + TCM + CM	1 (74)	RR 0.50 (0.05 to 5.28)	−1①	0	0	−1③	−1④	Very low

*First defecation time*								
AT + CM	3 (244)	SMD −2.05 (−2.39 to −1.72)	0	−1②	0	0	0	Moderate
AT	1 (60)	SMD −1.64 (−2.24 to −1.05)	0	0	0	−1③	0	Moderate
AT + TCM + CM	1 (387)	SMD −1.03 (−1.26 to −0.79)	−1①	0	0	0	−1④	Low

*First flatus time*								
AT + CM	7 (648)	SMD −2.66 (−3.82 to −1.50)	−1①	−1②	0	0	0	Low
AT	1 (60)	SMD −0.69 (−1.21 to −0.17)	0	0	0	−1③	0	Moderate
AT + TCM + CM	2 (461)	SMD −2.07 (−2.31 to −1.83)	−1①	0	0	0	−1④	Low

*First bowel sounds time*								
AT + CM	4 (402)	SMD −2.85 (−3.15 to −2.55)	−1①	−1②	0	0	0	Low
AT + TCM + CM	2 (461)	SMD −2.91 (−3.19 to −2.64)	−1①	0	0	0	−1④	Low

*Notes*. ①Most information is from the moderate risk studies, and there are major limitations. ②The size and direction of the effect size, the overlap of the confidence interval is small, the *p* value of the heterogeneity test is small, and the combined results of *I*^2^ value are large. ③The sample is insufficient. ④Few studies are included, and there may be a large publication bias.

**Table 5:** Adverse events in included studies.

Study (reference)	Sample size (A)/(B)	(A) Treatment group	(B) Control group	Adverse events
Liu and Zhang 2013 [37]	55/55	MA	CM (ondansetron)	A : none.
				B: 2 cases of dizziness, 3 cases with constipation, and 13 cases with extrapyramidal symptoms
Shen 2014 [39]	57/57	MA + (B)	CM (Metoclopramide)	A: 1 case of dizziness
				B: 3 cases of dizziness, 1 case with constipation, and 2 cases with extrapyramidal symptoms
Jing 2017 [40]	40/40	MA + (B)	CM	None
Xiao 2012 [43]	60/60	MA	CM	
			(fentanyl and morphine)	A: 1 case of dizziness
				B: 25 cases of PONV and 2 cases with hypotension

**Table 6:** Full-text articles excluded with reasons.

Full-text articles excluded	Reasons
Cai 2018^1^	Non-RCT
Pan 2017^2^	Non-RCT
Wang 2019^3^	Non-RCT
Shen et al. 2002^4^	Not acupuncture
Zhang et al. 2012^5^	Not acupuncture

References: ^1^Cai C. Clinical observation on the effect of warm acupuncture on the recovery of gastrointestinal function after cholecystectomy. Chinese and Foreign Medical Research. 2018; 16 (25):34–36. ^2^Pan D. Clinical observation on the recovery of gastrointestinal function after laparoscopic cholecystectomy in 60 patients with acupuncture. For All Health. 2017; 11 (10):165-166. ^3^Wang C. Effects of acupuncture at Zusanli and Hegu on gastrointestinal dysfunction after gallbladder stones. Xinjiang Medical University; 2019. ^4^Shen P, Xu Y, Jiang W, et al. Clinical study on acupoint electrical stimulation to promote recovery of gastrointestinal function after operation--A clinical data of 30 cases. Jiangsu Journal of Traditional Chinese Medicine. 2002; 23 (7):33-34. ^5^Zhang F, Li S, Li N. Effect of acupoint pulse electrical stimulation on intestinal function recovery after cholecystectomy. Today Nurse. 2012; 12:29-30.

**Table 7:** The PRISMA checklist about this SR.

Section/topic	#	Checklist item	Reported on page #
Title			
Title	1	Identify the report as a systematic review, meta-analysis, or both	1
Abstract			
Structured summary	2	Provide a structured summary including, as applicable, background; objectives; data sources; study eligibility criteria, participants, and interventions; study appraisal and synthesis methods; results; limitations; conclusions and implications of key findings; and systematic review registration number	1
Introduction			
Rationale	3	Describe the rationale for the review in the context of what is already known	2
Objectives	4	Provide an explicit statement of questions being addressed with reference to participants, interventions, comparisons, outcomes, and study design (PICOS)	2
Methods			
Protocol and registration	5	Indicate if a review protocol exists, if and where it can be accessed (e.g., Web address), and, if available, provide registration information including registration number	2
Eligibility criteria	6	Specify study characteristics (e.g., PICOS, length of follow-up) and report characteristics (e.g., years considered, language, and publication status) used as criteria for eligibility, giving rationale	3
Information sources	7	Describe all information sources (e.g., databases with dates of coverage and contact with study authors to identify additional studies) in the search and date last searched	2
Search	8	Present full electronic search strategy for at least one database, including any limits used, such that it could be repeated.	2-3
Study selection	9	State the process for selecting studies (i.e., screening, eligibility, included in systematic review, and, if applicable, included in the meta-analysis)	3
Data collection process	10	Describe method of data extraction from reports (e.g., piloted forms, independently, and in duplicate) and any processes for obtaining and confirming data from investigators	3-4
Data items	11	List and define all variables for which data were sought (e.g., PICOS and funding sources) and any assumptions and simplifications made	3-4
Risk of bias in individual studies	12	Describe methods used for assessing risk of bias of individual studies (including specification of whether this was done at the study or outcome level), and how this information is to be used in any data synthesis	4
Summary measures	13	State the principal summary measures (e.g., risk ratio and difference in means)	4
Synthesis of results	14	Describe the methods of handling data and combining results of studies, if done, including measures of consistency (e.g., I^2^) for each meta-analysis	4
Risk of bias across studies	15	Specify any assessment of risk of bias that may affect the cumulative evidence (e.g., publication bias and selective reporting within studies)	4
Additional analyses	16	Describe methods of additional analyses (e.g., sensitivity or subgroup analyses and meta-regression), if done, indicating which were prespecified	4
Results			
Study selection	17	Give numbers of studies screened, assessed for eligibility, and included in the review, with reasons for exclusions at each stage, ideally with a flow diagram	4
Study characteristics	18	For each study, present characteristics for which data were extracted (e.g., study size, PICOS, and follow-up period) and provide the citations	4-5
Risk of bias within studies	19	Present data on risk of bias of each study and, if available, any outcome level assessment (see item 12)	5
Results of individual studies	20	For all outcomes considered (benefits or harms), present, for each study: (a) simple summary data for each intervention group (b) effect estimates and confidence intervals, ideally with a forest plot	5-8
Synthesis of results	21	Present results of each meta-analysis done, including confidence intervals and measures of consistency	5-8
Risk of bias across studies	22	Present results of any assessment of risk of bias across studies (see Item 15)	8
Additional analysis	23	Give results of additional analyses, if done (e.g., sensitivity or subgroup analyses, meta-regression (see Item 16))	5-8
Discussion			
Summary of evidence	24	Summarize the main findings including the strength of evidence for each main outcome; consider their relevance to key groups (e.g., healthcare providers, users, and policy makers)	8
Limitations	25	Discuss limitations at study and outcome level (e.g., risk of bias), and at review-level (e.g., incomplete retrieval of identified research and reporting bias)	9
Conclusions	26	Provide a general interpretation of the results in the context of other evidence, and implications for future research	9-10
Funding			
Funding	27	Describe sources of funding for the systematic review and other support (e.g., supply of data); role of funders for the systematic review	10

Source: Moher D, Liberati A, Tetzlaff J, Altman DG, The PRISMA Group (2009). Preferred Reporting Items for Systematic Reviews and Meta-Analyses: The PRISMA statement. *PLoS Med* 6(6): e1000097. doi:10.1371/journal.pmed1000097. For more information, visit: http://www.prisma-statement.org.

## Data Availability

No data were used to support this study.

## Appendix

### (A). Full-Text Articles Excluded with Reasons

Full-text articles excluded with reasons are given in Table 6.

### (B). PRISMA-2009 Checklist

PRISMA-2009 checklist is given in Table 7.
